# Using an mHealth approach to collect patient-generated health data for predicting adverse health outcomes among adult survivors of childhood cancer

**DOI:** 10.3389/fonc.2024.1374403

**Published:** 2024-05-10

**Authors:** Kristen E. Howell, Marian Shaw, Aimee K. Santucci, Kristy Rodgers, Izeris Ortiz Rodriguez, Danah Taha, Sara Laclair, Carol Wolder, Christie Cooper, Wonjong Moon, Christopher Vukadinovich, Matthew J. Erhardt, Shannon M. Dean, Gregory T. Armstrong, Kirsten K. Ness, Melissa M. Hudson, Yutaka Yasui, I-Chan Huang

**Affiliations:** ^1^ Department of Epidemiology and Biostatistics, Texas A&M University, College Station, TX, United States; ^2^ Department of Epidemiology and Cancer Control, St. Jude Children’s Research Hospital, Memphis, TN, United States; ^3^ Department of Oncology, St. Jude Children’s Research Hospital, Memphis, TN, United States; ^4^ Department of Pediatric Medicine, St. Jude Children’s Research Hospital, Memphis, TN, United States

**Keywords:** childhood cancer survivors, electronic health record, late effects, machine learning, mHealth, patient-generated health data

## Abstract

**Introduction:**

Cancer therapies predispose childhood cancer survivors to various treatment-related late effects, which contribute to a higher symptom burden, chronic health conditions (CHCs), and premature mortality. Regular monitoring of symptoms between clinic visits is useful for timely medical consultation and interventions that can improve quality of life (QOL). The Health Share Study aims to utilize mHealth to collect patient-generated health data (PGHD; daily symptoms, momentary physical health status) and develop survivor-specific risk prediction scores for mitigating adverse health outcomes including poor QOL and emergency room admissions. These personalized risk scores will be integrated into the hospital-based electronic health record (EHR) system to facilitate clinician communications with survivors for timely management of late effects.

**Methods:**

This prospective study will recruit 600 adult survivors of childhood cancer from the St. Jude Lifetime Cohort study. Data collection include 20 daily symptoms via a smartphone, objective physical health data (physical activity intensity, sleep performance, and biometric data including resting heart rate, heart rate variability, oxygen saturation, and physical stress) via a wearable activity monitor, patient-reported outcomes (poor QOL, unplanned healthcare utilization) via a smartphone, and clinically ascertained outcomes (physical performance deficits, onset of/worsening CHCs) assessed in the survivorship clinic. Participants will complete health surveys and physical/functional assessments in the clinic at baseline, 2) report daily symptoms, wear an activity monitor, measure blood pressure at home over 4 months, and 3) complete health surveys and physical/functional assessments in the clinic 1 and 2 years from the baseline. Socio-demographic and clinical data abstracted from the EHR will be included in the analysis. We will invite 20 cancer survivors to investigate suitable formats to display predicted risk information on a dashboard and 10 clinicians to suggest evidence-based risk management strategies for adverse health outcomes.

**Analysis:**

Machine and statistical learning will be used in prediction modeling. Both approaches can handle a large number of predictors, including longitudinal patterns of daily symptoms/other PGHD, along with cancer treatments and socio-demographics.

**Conclusion:**

The individualized risk prediction scores and added communications between providers and survivors have the potential to improve survivorship care and outcomes by identifying early clinical presentations of adverse events.

## Introduction

The 5-year survival of childhood cancer exceeds 85% today, with the population of childhood cancer survivors estimated to reach approximately 580,000 by 2040 in the United States (1). However, cancer therapies predispose survivors to different late effects, including physical/psychological sequelae, subsequent neoplasms, and premature death (2–5). It has been previously estimated that by age 50 years, survivors develop a mean of 17.1 chronic health conditions, of which 4.7 are severe, disabling, or life-threatening (6). Additionally, childhood cancer survivors often endorse poor patient-reported outcomes (PROs), including more symptoms and lower quality of life (QOL), as compared to sibling controls or the general population (7–9). Symptomology, defined as a perceived abnormal physical, emotional, or somatic state, is a meaningful prognostic marker of survivorship. Over 75% of survivors have multiple symptoms (10) and over 50% of survivors have moderate or high symptom burden (9). Among cancer survivors, studies have observed the increase of symptoms over 25 years, and those having more symptoms have greater impaired QOL (8, 10, 11). This increasing symptom burden in survivors is associated with higher CHC burden, including cardiac, respiratory, neurologic, and musculoskeletal disorders, and premature mortality compared to the general population (11, 12).

Perception of symptomatic abnormality is subjective and dynamic and may fluctuate from moment to moment or day to day. A randomized trial found that collecting symptom and QOL data weekly from active adult cancer patients increased patient-doctor conversations about treatment regimens, leading to improved QOL in the following six months, fewer emergency room visits in the following three years, and higher survival rates compared to a standard care group (13–15). Therefore, timely monitoring of patient-reported symptoms may be useful in identifying individuals with declining health status who may benefit from further clinical assessment and intervention (16, 17).

In addition to symptomology, other patient-generated health data (PGHD), including physical activity intensity, sleep/lifestyle behavior, and biometric data (e.g., resting heart rate, heart rate variability, oxygen saturation), have a unique value in predicting future health (18, 19). Similar to the concept of PGHD, other research introduces the term “Personal Health Records” (PHR) (20), which specifically denotes health databases controlled and maintained by patients. Many PGHD or PHR platforms often enable the consolidation of data from various sources, such as wearable technology or digital devices, allowing patients to track their health conditions effectively. Evidence suggests that fewer step counts per day, more fragmented physical activity (e.g., bouts lasting <5 minutes), and suboptimal sleep duration or inefficient sleep increase all-cause mortality (21–23). Alternatively, performing more step counts per minute or stairs per day contributes to better QOL (24). Leveraging mobile health (mHealth) technology, including smartphones to assess daily symptoms, augmented with wearable devices/sensors to assess momentary PGHD from typical daily activities, provides promising opportunities to improve risk prediction of impaired health status and to offer early interventions (25–27).

Implementing symptom/PGHD-based risk prediction models may improve current survivorship care by early detection and intervention. The Children’s Oncology Group (COG) Long-Term Follow-Up Guidelines, provide surveillance recommendations for the detection of exposure-specific adverse health outcomes (28). The COG Guidelines are based on empirical evidence and not on regular assessment of symptom prevalence or severity and/or changes in other health biometrics over time. Findings from a recent study support that regular symptom assessments facilitate adult cancer diagnosis at earlier stages (29). Currently, remote symptom/PGHD monitoring is not utilized regularly in survivorship care (30–32). Therefore, developing a PGHD-based survivorship risk prediction model can enhance COG-recommended surveillance by supplementing observations in clinical encounters, informing clinicians about risk classification for adverse outcomes, and triggering communication with survivors for self-management.

Healthcare providers have expressed concerns with PGHD utilization, including the added burden on both patients and clinicians (19, 33, 34), technical and legal issues such as data security (35), and the usability, accuracy, and completeness of the data (36, 37). To address these concerns, the use of secure servers and messaging systems has been incorporated into PGHD systems (34, 38). Additionally, care coordinators (i.e., nurses) can offer training to both patients and caregivers on the suitable use of technologies as well as monitor and manage large amounts of PGHD data (34, 39). The utilization of PGHD has been on the rise with the emergence of novel technology; however, it is uncommon for PGHD to be integrated into the electronic health record (EHR) (19, 40). Integration of PGHD into the EHR creates an opportunity to enhance the clinical workflow, facilitating patient-clinician communication and clinical decision-making. To successfully integrate PGHD into the EHR, interoperability of the data platforms must be addressed.

The overarching goal of the present study is to enable regular monitoring of PGHD, including daily symptoms, momentary physical activity intensity, energy expenditure, sleep performance, and heart rate variability, and utilize these data to develop survivor-specific risk prediction models for adverse health outcomes of childhood cancer. Further, we aim to calculate risk scores and integrate this information into the existing EHR to improve survivorship care and outcomes. This study is an R01 grant funded by the US National Cancer Institute (NCI) to address NCI’s provocative questions (RFA-CA-20-004), “What methods can be developed to integrate patient-generated health data into electronic health records?” (41).

## Methods

### Study objectives, specific aims, and hypotheses

**Figure 1:**
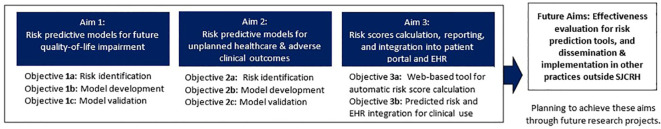
Overview of aims and corresponding research activities.

The primary aim (Aim 1) of this study is to leverage a mHealth platform to collect and integrate PGHD data and develop/validate risk prediction models for future QOL outcomes using longitudinal, dynamic data (**Figure 1**). Aim 1 will be approached by (1) investigating the variability of PGHD within and between survivors with special attention to their temporal change patterns, (2) assessing associations and temporal patterns of the mHealth-collected PGHD while considering clinical and socio-demographic factors, (3) developing risk prediction models for future QOL outcomes using training data with cross-validation and evaluate model performance using independent test data, and (4) establishing personalized risk prediction models for adverse health outcomes for potential use within clinical settings. The second aim (Aim 2) will develop/validate risk prediction models and establish personalized risk prediction scores for additional health outcomes such as unplanned healthcare utilization, objectively assessed physical performance deficits, and the onset of clinically ascertained chronic health conditions using the same approach as described in Aim 1. The third aim (Aim 3) will create a web-based tool to calculate and report personalized outcome-specific risks and facilitate the integration of risk scores into the survivor’s patient portal (i.e., a secured patient website to display participants’ health risks and a mechanism to communicate with a healthcare provider about the management of treatment-related late effects) and hospital’s EHR for potential future use/evaluation in clinical management.

### Study design

**Figure 2:**
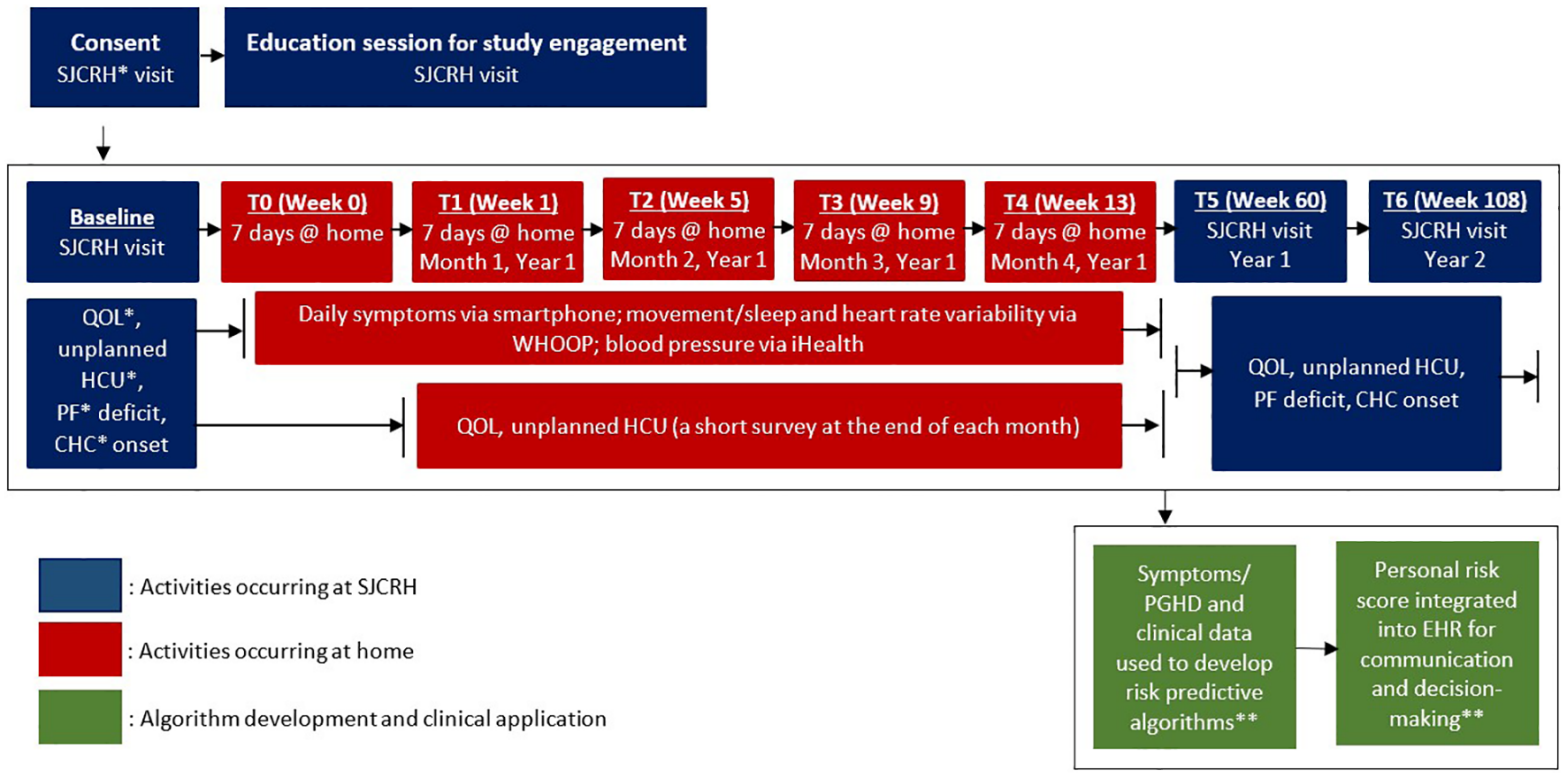
Data collection from study participants (A.ims 1 and 2). *SJCRH: St. Jude Children’s Research Hospital; QOL: quality of life; HCU: healthcare utilization; PF: physical performance; CHC: chronic health conditions. ** See **Figure 3** for integrating symptoms/PGHD into patient portal/EHR for survivorship care and research.

This study uses a prospective design to collect symptoms and other PGHD as risk factors for subsequent adverse health outcomes. Data collection from participants include daily symptoms via a smartphone, other PGHD (physical activity intensity, sleep performance, biometric data, including resting heart rate, heart rate variability, oxygen saturation, skin temperature, physical stress) via a wearable activity monitor, patient-reported outcomes (impaired QOL, unplanned healthcare utilization) via a smartphone, and clinically ascertained outcomes (physical performance deficits, onset of/worsening chronic health conditions) assessed in the survivorship clinic at St. Jude Children’s Research Hospital (SJCRH). The research timeline includes the following 8 time points over 2 years (**Figure 2**). Research activities involve baseline for assessing initial PROs and clinical outcomes in the survivorship clinic; T0 (7 days in week 0) for testing the study devices and T1 (7 days in week 1), T2 (7 days in week 5), T3 (7 days in week 9), and T4 (7 days in week 13) for collecting symptoms/PGHD in non-clinical, daily-living settings; T5 (week 60) and T6 (week 108) for collecting PROs and clinical outcomes in the survivorship clinic.

### Participants and recruitment

We aim to enroll 600 cancer survivors (Aims 1 & 2) from the current 6,005 active participants of the St Jude Lifetime Cohort Study (SJLIFE) (6, 42, 43). SJLIFE eligibility criteria include: diagnosis of childhood cancer; treatment at SJCRH between 1962-2012; and survival ≥ 5 years post-diagnosis of pediatric cancer/malignancy (43). Through the SJLIFE protocol, survivors are invited to return to SJCRH at least once every 5 years for medical evaluations and assessments of neurocognitive function, physical performance status, and patient-reported outcomes (43).

Inclusion criteria for the present study include ≥18 years of age at the time of enrollment, ≥5 years from initial diagnosis of pediatric cancer/malignancy, currently not receiving cancer therapies, and access to a web-enabled smartphone. Exclusion criteria include unable to communicate in English, unable to consent for self, and being currently pregnant or planning to become pregnant in the next two years.

We will invite an additional 20 adult survivors of childhood cancer survivors from SJLIFE to explore their perspectives about suitable formats to display predicted risk information on a dashboard (i.e., a patient-clinician interface embedded within the EHR), as well as 10 clinicians (5 survivorship and 5 primary care clinicians) from SJCRH and affiliates to suggest evidence-based risk management strategies by considering PGHD-based risk profiles and clinical/treatment data. Our criteria for recruiting clinical stakeholders include attending pediatric oncologists, advanced practice providers, and academic onco-primary care providers who have > 3 years of experience in survivorship care.

### Study procedures

**Figure 3:**
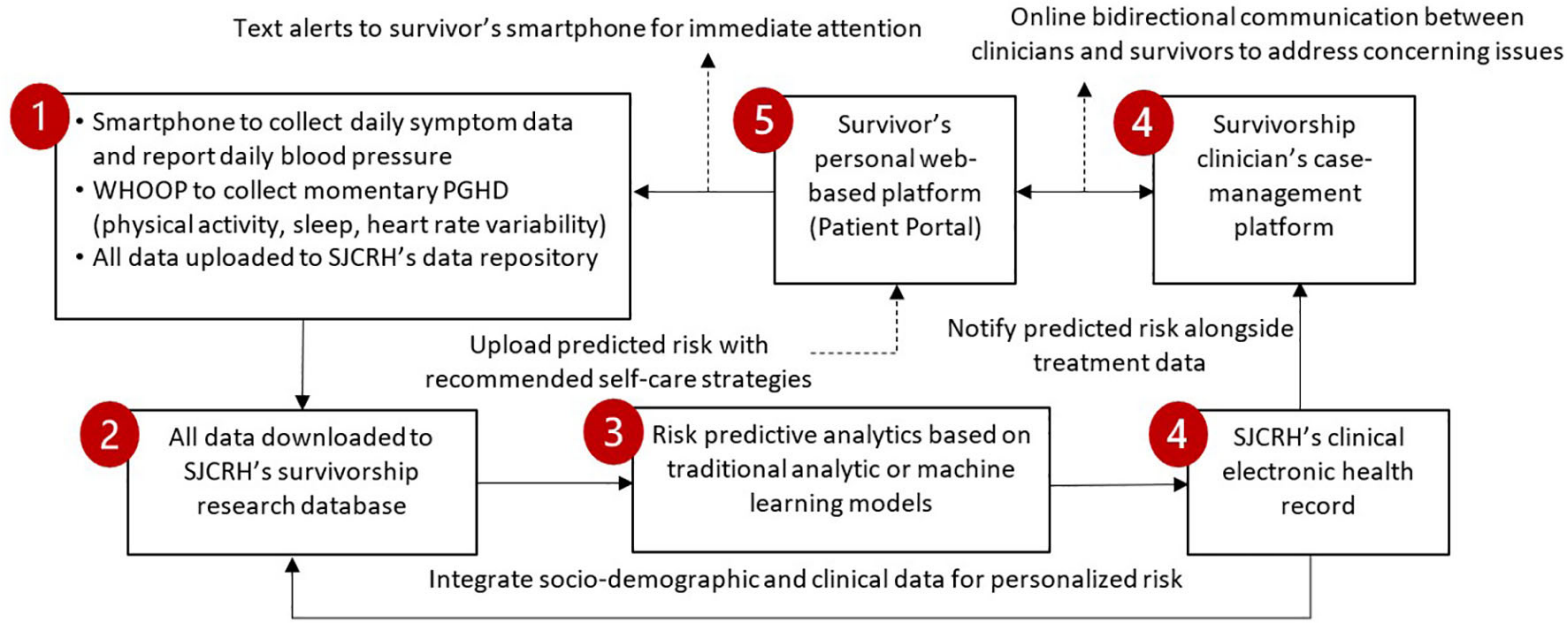
Workflow that integrates symptoms/PGHD into patient portal/EHR for survivorship care and research.

**Figure 3** shows a 5-step workflow that integrates symptoms/PGHD into the patient portal/EHR for survivorship care and research. In Steps 1 and 2, we will use a smartphone to collect daily symptoms, a Bluetooth-enabled cuff to measure blood pressure data and a wearable device, WHOOP (see the section “WHOOP 4.0” below), to collect momentary PGHD. Data will be uploaded to SJCRH’s data repository. The automated messaging workflow for collecting daily symptom data has been pilot-tested and demonstrated a mean of >90% adherence rate each week for daily symptom reporting (44). In Step 3, we will use informatics technology to automatically calculate personalized risk scores of adverse outcomes based on validated machine/statistical models built in Aims 1 and 2 to indicate each survivor’s risk based on their features/patterns of risk predictors. In Step 4, we will send interpretable predicted scores of adverse outcomes, augmented with decision support for self-care, within the EHR to the clinician. In Step 5, we will post the interpretable predicted risk of adverse outcomes to the patient portal and send a notification to the survivor’s mobile device. Steps 4 and 5 are intended for promoting the initiation of clinician/survivor bidirectional communication to discuss a care plan or schedule an appointment.

#### Patient interface system

We will use a HIPPA-compliant patient interface system (i.e., patient portal) for collecting PGHD through smartphones and wearable devices, depositing the analytic output, and integrating risk prediction information into the patient portal and the hospital’s EHR. The interface system is a patient-centered platform for clinical engagement as it can organize the momentary PGHD flows via a real-time data collection, storage, and transmission system; send reminders by text messaging and email to increase study compliance and decrease data missingness; and arrange automatic incentive payment delivery. Study participants will receive daily messages from the patient interface system to remind them to complete the daily study activities, such as reporting their daily symptom log and blood pressure measures (**Figure 2**).

The daily symptom log includes the following 20 symptoms: irritability, anxiety, depression, fatigue or feeling weak, difficulty falling asleep or staying asleep at night, sleepy during the day, poor memory, lack of concentration, shortness of breath, chest pain during physical exercise, numbness or tingling, problem with balance, headache, bodily pain, swelling, cramps, constipation, diarrhea, lack of appetite, and poor concentration. Previous studies have demonstrated that these 20 symptoms are highly prevalent and clinically meaningful among childhood cancer survivors (9, 10). Participants will be instructed to complete this log daily for four consecutive months by selecting which symptoms, if any, they have experienced in the last 24 hours. The daily symptom log takes 1-2 minutes to complete. For each symptom that the participant selects as “present”, they will be asked the severity of the symptom on a five-point Likert scale (very mild, mild, moderate, severe, and very severe) and how much the symptom interfered with their daily activities on a five-point Likert scale (not at all, a little bit, moderately, quite a bit, and very much).

We will conduct full-scale surveys through the patient interface system at three of the 8 major time points (baseline, T5, and T6) to collect PROs for use as outcome variables (QOL, healthcare utilization, and health-related employment) or covariates (social support and coping behavior). These PROs include generic and cancer-specific QOL using the NIH Patient-Reported Outcomes Measurement Information System 43-Profile (PROMIS-43) (45), Quality of Life in Adult Cancer Survivors (QLACS) (46), and EuroQol-5D (EQ-5D) (47), social support using the Duke-UNC Functional Social Support Questionnaire (FSSQ) (48), coping behavior using the Brief COPE (49), and some items selected from the U.S. Medical Expenditure Panel Survey (MEPS) measuring healthcare utilization and health-related employment (50). Additionally, we will conduct short-scale surveys to collect key PROs of interest based on NIH PROMIS-43 and EQ-5D from the last day of T1, T2, T3, and T4 (**Table 1**).

#### Whoop 4.0

**Figure 4:**
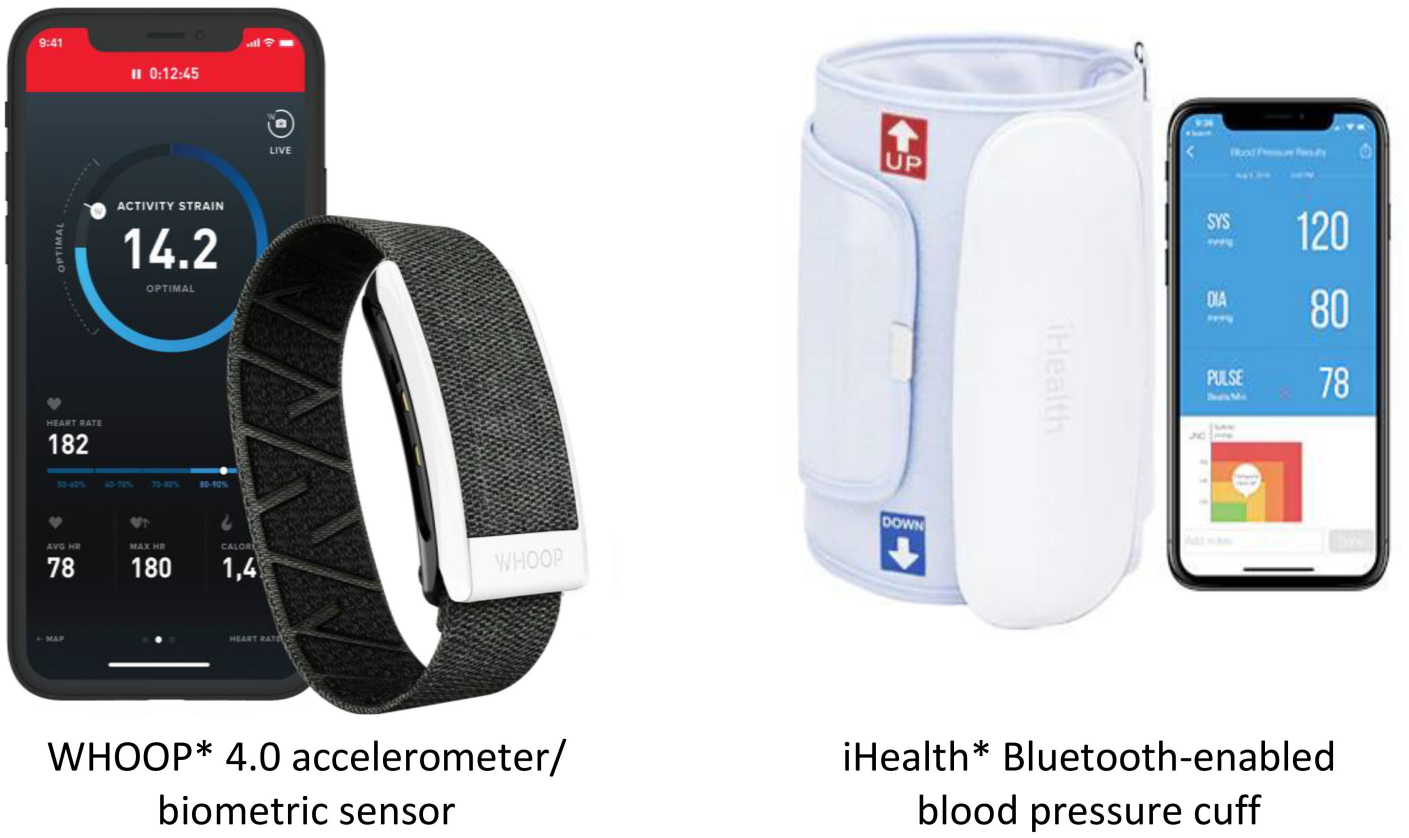
WHOOP accelerometer/biometric sensor and iHealth Bluetooth-enabled blood pressure cuff. *Sources of images. WHOOP Inc (https://www.whoop.com/us/en/). iHealth Labs Inc (https://ihealthlabs.com/).

The WHOOP (Strap 4.0, WHOOP Inc, Boston, Massachusetts) is a Bluetooth-enabled, wrist-wearable device that collects measures of physical activity, energy expenditure, sleep performance, oxygen saturation (SpO2), respiratory rate, skin temperature, physiological stress, resting heart rate, and heart rate variability using five LEDs and four photodiodes (**Figure 4**) (51). The WHOOP device has been previously validated in its ability to measure heart rate and sleep performance (52–55). Participants will be instructed to wear the WHOOP 4.0 device for at least 20 hours per day for four consecutive months. Data from the WHOOP device will be uploaded continuously (as long as the participants’ device is within Bluetooth coverage range and the application is open) and stored securely on WHOOP’s cloud, not the participants’ smartphone. Data can be stored on the WHOOP device for up to 2 weeks if it is not connected to the participants’ smartphone. Data will be sent to the study’s research server daily for compliance monitoring, data management, and statistical analyses. The study team also receives a daily compliance report and a weekly summary report via email from the WHOOP Inc. The daily compliance report informs when the device was last synced to WHOOP’s server and how many hours the device was off the participants’ wrists. The weekly summary report will be sent twice per week and includes the previous seven days of participants’ recovery (hours in bed, hours of sleep, sleep efficiency, sleep disturbances), strain (strain score, average heart rate), and workouts (activity type, activity duration, max heart rate, average heart rate, calories burned). Additionally, the WHOOP company has developed an application programming interface (API) to deliver the participants’ daily raw data to the study’s research server, which includes nearly second-by-second recordings of heart rate, accelerometer axes, and skin temperature.

#### iHealth blood pressure cuff

The iHealth blood pressure cuff is a Bluetooth-enabled device that has been clinically validated and cleared by the U.S. Food and Drug Administration (FDA) for monitoring blood pressure (**Figure 4**) (56, 57). Participants will be instructed to take their blood pressure twice daily in one sitting (one minute apart) over four consecutive months (one week per month is required and the other three weeks are optional). Participants will be instructed to fill out a Blood Pressure log on the patient interface system immediately following the measurement. The Health Share team will download the data from the participants’ de-identified accounts on the iHealth Cloud monthly to the study’s research server for data management and statistical analysis.

### Data collection and analysis

#### Study timeline

##### On campus activities

All SJLIFE participants who are scheduled to return to SJCRH for a comprehensive medical assessment (i.e., SJLIFE visit) are potentially eligible for participation in the Health Share Study (42). Before their visit, an SJLIFE visit coordinator calls the participants and briefly informs them about the Health Share Study to gauge their interest. If they express interest in participating in the Health Share Study, a consent appointment and an education appointment will be scheduled during their SJLIFE visit. During the 30-minute consent appointment, a Health Share team member will inform the potentially eligible participant about the study activities and obtain informed consent. Following consent, the participant will attend a 90-minute education session during which a Health Share team member will help the participant set up the study devices (i.e., the WHOOP and iHealth apps on the participants’ smartphone, WHOOP device, and iHealth Bluetooth-enabled blood pressure measure) and provide detailed instructions on the study activities. The participant will also complete a baseline survey, administered through the patient interface system, which includes the NIH PROMIS-43, QLACS, EQ-5D, FSSQ, Brief COPE, and healthcare utilization and health-related employment questionnaires **Table 1**.

**Table 1:** Study activities completed at each time point.

Study Activities	Time point				
	Baseline	Feasibility Week (T0)	Months 1-4 (T1-T4)*	Week 60 (T5)	Week 108 (T6)
Consent	X				
NIH PROMIS-43, EQ-5D, healthcare utilization and health-related employment	X		X	X	X
QLACS, Duke-UNC FSSQ, and Brief COPE	X			X	X
SJLIFE standard assessments - Clinical assessments - Physical performance tests	X			X	X
Daily symptom report		X	X		
Momentary physical activity monitor via wearable		X	X		
Daily blood pressure assessment		X	X		

COPE, Coping Orientation to Problems Experienced; EQ-5D, Europe Quality of Life; FSSQ; Functional Social Support Questionnaire; NIH, National Institutes of Health; PROMIS, Patient Reported Outcomes Measurement Information System; QLACS, Quality of Life in Adult Cancer Survivors; SJLIFE, St Jude Lifetime Cohort Study; T, time point; UNC, University of North Carolina.

SJLIFE Standard Assessments include: Home survey, health habits survey, psychosocial survey, men’s/women’s/adolescent health survey, Block food frequency questionnaire, core health evaluation, functional assessment, echocardiogram, pulmonary function testing, audiology evaluation, ophthalmology, social work, dual x-ray absorptiometry scan, quantitative computed tomography scan, comprehensive blood panel, lipid panel, and urinalysis.

*The first week of each month is required and the other three weeks are optional.

While on campus, as part of SJLIFE, participants will complete a modular health questionnaire, and undergo comprehensive medical assessments and physical performance assessments (42). All participants will undergo core battery medical evaluations: blood pressure measurement; physical examination including a detailed neurologic assessment; and laboratory tests for complete blood cell count, metabolic panel, fasting lipid profile, insulin, hemoglobin A1c, thyroid and gonadal function, and urinalysis. Participants will also receive a systematic evaluation of organ function including: 1- and 2-lead electrocardiography; echocardiography, pulmonary function testing, audiogram, ophthalmologic evaluation, and bone mineral density testing. Using the medical assessment data, individual chronic health conditions will be grouped by major organ systems (**Table 2**). According to a modified version of the National Cancer Institute’s Common Terminology Criteria for Adverse Events (CTCAE) Version 4.03 for childhood cancer survivors (58), the severity of each condition is assigned as none (Grade 0), mild (Grade 1), moderate (Grade 2), serious/disabling (Grade 3), and life-threatening (Grade 4).

**Table 2:** Adverse health outcomes through medical assessment and physical performance tests.

Groups of CHCs	Individual CHCs identified through medical assessment
Vascular	• Hypercholesterolemia • Hypertriglyceridemia • Hypertension
Cardiac	• Atrioventricular Heart Block • Cardiac Dysrhythmia • Cardiomyopathy • Conduction Abnormality • Heart Valve Disorder • Myocardial Infarction • Prolonged QT Interval • Pulmonary Hypertension • Right ventricular systolic dysfunction
Endocrine	• Adrenal insufficiency • Adult GHD • Abnormal glucose metabolism • Hyperthyroidism • Hypothyroidism Central • Hypothyroidism Primary
Respiratory	• Chronic obstructive pulmonary disease • Obstructive pulmonary deficit • Obstructive sleep apnea • Pulmonary diffusion deficits • Pulmonary embolism • Restrictive pulmonary deficit
Neurologic	• CV disease • Cerebellar dysfunction • Cerebrovascular accident • Cerebrovascular disease • Cranial nerve disorder • Intracranial hemorrhage • Movement disorders • Multiple sclerosis • Peripheral motor neuropathy • Peripheral sensory neuropathy • Seizure
Musculoskeletal	• BMD below expected range for age and sex • Hernia • Intervertebral disc disorder • Kyphosis • Osteonecrosis • Scoliosis
Reproductive	• Erectile dysfunction • Hypogonadism Central • Primary ovarian failure • Leydig cell failure
Domain of physical performance	Tests to identify physical performance deficits
Cardio-pulmonary fitness	Six-minute walk; cardiopulmonary exercise testing
Flexibility	Sit-and-reach test (Flex- tester); passive ankle range of motion
Mobility	Timed up-and-go test
Muscular strength & endurance	Isometric grip strength; isokinetic knee extension and ankle dorsiflexion (Biodex System 4)
Balance & sensory integrity	Computerized dynamic posturography (SMART EquiTest)

HCs, Chronic health conditions.

Physical performance assessments include flexibility, mobility, balance and sensory integrality, muscle strength and endurance, and cardiopulmonary fitness tests. Tests will be conducted at SJCRH’s Human Performance Lab by licensed/certified examiners. **Table 2** lists the types of medical and physical performance tests to be conducted, and these tests are based on standardized tools with good reliability and clinical validity.

The same participants will be invited to return to SJCRH one and two years (T5 and T6) after the initial baseline visit. During the follow-up visits, participants will undergo a similar research activity to the baseline, including medical and neuromuscular assessments as well as PRO surveys (**Tables 1**, **2**). Participants are provided travel support and accommodations, and compensated for each day that they are on campus during the baseline and follow-up visits for their time and efforts to complete these evaluations.

##### At-home activities

Following the baseline visit, the participant will return home and begin the testing week. The purpose of the testing week is to test whether all devices are implemented properly in the home setting, to identify barriers to providing PGHD on a regular timeline, and to find its solution to facilitate the data collection. Participants will be asked to wear their WHOOP for five days, complete one symptom log, and measure their blood pressure twice in one setting, with one minute between the two measurements. Following the completion of the testing week activities, participants will receive a $10 gift card for their time and efforts to complete these activities.

After completing the testing week, participants will begin a four-month research period at home where they are asked to wear WHOOP 4.0, measure their blood pressure, and report their daily symptoms for at least five out of seven days each week. The first week of each month is considered “required”, and the participant will earn a gift card for completing the required study activities. The following three weeks of each month are considered “optional”, and the participant will earn a gift card for completing the study activities each week. At the end of each required week, participants will be asked to complete a weekly impact survey, which includes the NIH PROMIS-43, EQ-5D, and healthcare utilization and health-related employment questionnaires (**Table 1**).

Participants will access the daily activities through the patient interface system. During the baseline visit, each participant will be given a unique account for the portal, where they can view the daily assignments. The patient interface system has been programmed to automatically roll over to the next day’s assignments each day. Additionally, the patient interface system will be used to keep track of participant compliance with study activities and distribute gift codes if the participant meets the study’s incentive requirements each week. The portal holds the participant’s contact information and distributes emails and text messages every day to inform the participant of that day’s study activities and when they have received an incentive. Informational flyers will be also posted on the patient interface system to help inform participants on how to use the study devices and complete the daily activities.

#### Designing a user-friendly dashboard and a rapid decision/discussion support tool

##### Developing a user-friendly dashboard to display personalized risk of adverse health outcomes

We will assess 20 survivors for their preferences/needs through 3 focus group sessions. Each session will include 6-7 survivors of a similar education level (high school, college, graduate degree), age (<45, ≥45 years), and sex. During each session, the session leaders will share content and format examples for displaying personal predicted risk, brainstorm new ideas for data visualization, and rank the importance by a nominal group process. We will employ standard methods to create the interview guide, beginning with broad questions aimed at understanding survivors’ overall preferences for reviewing their symptom/PGHD data, followed by discussing various formats for presenting the data. We plan to draft different designs of the platform and ask whether survivors prefer different styles of data presentation to display their symptom/PGHD data, such as symptoms over time (i.e., bar chart vs line graph). We will ask if survivors prefer to see their symptom/PGHD data displayed month by month or aggregately. We will also determine the best way to display the personal risk of adverse health outcomes in a manner that the survivor will understand.

Based on these rankings, we will share candidate dashboard designs with 10 clinicians via 30-minute semi-structured interviews. We will collect comments on the contents, metrics, and formats in visualizing predicted risk for survivorship care. Each focus group session and interview will be audio-recorded, transcribed, and analyzed using the standard thematic methods (59). Our informatics team will create a low-fidelity prototype to illustrate risk prediction information for each survivor over time and compare the results of individual survivors to age/sex-matched survivors.

##### Developing a rapid decision/discussion support tool to facilitate risk management

Our aim is to set up an automatic predictive analytic to be performed to estimate risk scores of specific adverse outcomes for each survivor. We will use the following rule to categorize personal predicted risk and display information alongside personal risk factors relevant to adverse health outcomes on the dashboard: low (<1.2-fold the general population risk), average (1.2-1.5-fold risk), moderate (1.5-3.0-fold risk), and high (>3.0-fold risk) (60). The same 10 clinicians who engage in the dashboard development will be invited to take part in developing self-management guidelines based on extant literature with clinical consensus using a modified online Delphi process. We will review the literature and practice guidelines, summarize self-care strategies for improving symptoms and lifestyle factors, and provide a summary to the 10 clinicians. In round 1 of the Delphi process, the clinicians will rate the importance of each intervention for managing specific risk factors on a 1-9 Likert scale (1=least/9=most important). Results will be re-evaluated by the same clinicians in round 2. Self-care strategies rated by scores 7-9 for ≥80% of the clinicians will be deemed sufficient for use.

#### Integration of PGHD into EHR

Following the data collection and analyses, interpretable predicted scores of adverse outcomes based on the algorithms built in Aims 1 and 2 will be reported to a single dashboard via an integrated process within the EHR using a Central Cancer Survivorship Platform (CCSP) design (**Figure 3**). The CCSP design will be used to integrate symptoms/PGHD collection, automatic predictive analytics, and dashboard-based decision support for childhood cancer survivors and their healthcare providers. The output will be delivered to the EHR and survivors’ smartphones via the patient interface system. The integration process will involve the following steps (**Figure 3**):

- **Step 1:** use the patient interface system on smartphones to collect symptom data, the WHOOP wearable to collect activity/sleep data and other biometric data, and the iHealth meter to collect blood pressure data, and upload all data to SJCRH’s data repository using each device’s API or comparable programs;
- **Step 2:** download all data from the data repository to SJCRH’s survivorship research database for data cleaning, and integrate symptoms/PGHD with socio-demographic, diagnosis, and treatment data stored on SJCRH’s EHR;
- **Step 3:** use machine/statistical learning techniques to automatically calculate personalized risk scores of adverse outcomes based on the algorithms built in Aims 1 & 2, and each survivor’s features/patterns of risk predictors;
- **Step 4:** send interpretable predicted scores of adverse outcomes in an explicit form (e.g., discrete data field), augmented with decision support for self-care, within the EHR to clinicians and
- **Step 5:** post interpretable predicted risk of adverse outcomes to the patient portal and send a notification text to survivors’ mobile devices.

#### Data analysis plan

##### Missing data handling

Missing daily symptom/PGHD will be replaced by a forward-filling- then-backward-filling strategy, i.e., replaced by the value of the previous closest day, and if unavailable, the subsequent closest day.

##### Two approaches to prediction modeling building

###### Statistical learning by generalized linear models with elastic net regularization

For better interpretability, we will focus on manually-engineered patterns and predictor variables with Elastic Net, i.e., generalized linear models (GLM) of the form (E[Yi]) = (offset) + Zi^t^α + Xi^t^β + (Zi, Xi)^t^γ with regularization with a convex combination of L1 and L2 penalties and hyper parameters selected by cross-validation, where for ([A] piecewise exponential models for onset of/worsening chronic health conditions (Aim 2), [B] general linear models for QOL scores/changes (Aim 1) and human performance deficits/changes (Aim 2), and [C] logistic regression models for unplanned care utilization (Aim 2), respectively), h is the link function ([A] log, [B] identity, [C] logit), i denotes ([A] a time segment of a subject’s follow-up, [B] a subject, [C] a subject), Yi is the outcome random variable ([A] Poisson event count, [B] Gaussian continuous measurement, [C] Bernoulli presence/absence), “offset” is ([A] log person time at risk, [B] 0, [C] 0), Zi is a vector of symptoms and other PGHD, and Xi is a vector of clinical, socio-demographic predictors including sex, with (Zi, Xi) denoting interaction terms, and α, β, γ are parameter vectors. Additional parameters for the regularization are selected by cross-validation of the training data.

###### Machine learning approach

We will employ the Long Short-Term Memory (LSTM) Model of Recurrent Neural Network (RNN) without requiring the *a priori* pattern generation. LSTM was chosen because it is capable of handling complex interactions of high-dimensional time- indexed predictors, without *a priori* hypotheses of their existence/forms, such as interactions of 4-month x 7-day/month longitudinal patterns of multi-symptoms and PGHD and clinical/socio-demographic predictors. The architecture of an LSTM unit includes “memory” and “gates” to keep and throw away time-indexed predictors over time, where the input to each unit consists of: (M) “memory” from the previous unit; (I) PGHD of the current day/month, along with any other time-independent predictors (i.e., clinical and socio-demographic variables); and (O) “output” of the previous unit. Specifically, each unit takes these 3 inputs and uses 3 gates to create the unit’s “memory” and “output”: 1) “input gate” controlling what/how much new information from (I) and (O) to use in the current unit; 2) “forget gate” controlling what information to throw away from (I), (M), and (O); and 3) “output gate” controlling what/how much the final output of the unit should be from (I), (M) and (O). At the final layer, we will pool the outputs of all LSTM units from the 7 days/month x 4 months, followed by an activation function to squash the pooled prediction into the proper range, for example, the linear for QOL scores and the sigmoid for unplanned care utilization. The LSTM units’ gates use logistic and hyperbolic tangent functions to perform their jobs. A variant of gradient descent, Adam optimization, will be used for training LSTM. We will use a random search with cross-validation to select optimal hyper-parameters including several hidden layers, penalization rate, batch size, epoch and learning rate.

### Training/testing sets, nested cross-validation, and prediction performance measures

A stratified randomization of N=600 participants will be used to select N=500 as the training dataset and N=100 as the testing dataset, stratified by sex, race/ethnicity, childhood cancer type, era of diagnosis, age at the baseline of this study (10-year groups), and baseline chronic health conditions burden (high/moderate/low). The sample sizes of 500:100 was determined by assessing power for identifying associated factors in predicting an outcome in the training dataset and precision of sensitivity and specificity with the testing dataset. The minimum detectable hazard ratios with N=500, 80% power, and Type I error probability of 0.05, using the power calculation approach for the proposed time-to-event analysis, was 2.55 or smaller unless the outcome’s cumulative incidence is less than 10% and the predictor’s prevalence is less than 25%. This indicated good power for a wide range of moderately predictive predictors. With the testing data of 100 subjects, based on the precision of area under the curve (AUC) from the equivalence to Wilcoxon statistic, the margin of error of AUC estimation for underlying AUC of 0.90 is 0.12, 0.09, and 0.07 for 10%, 20%, and 30% cumulative incidence of CHCs. For AUC of 0.80, it is 0.15, 0.11, and 0.09, providing sufficient precision in testing data.

We will only use the N=500 training dataset to build prediction models with a nested cross-validation which allows the evaluation of prediction performance along with the selection of hyper-parameters. We will set aside the N=100 testing dataset until the final models have completely been developed with the training dataset and are ready for validation with a single-time unbiased evaluation of their prediction performance. Prediction performance will be assessed by Mean Squared Errors when the outcome Yi is a Gaussian continuous measurement, by receiver operating characteristic curves and its AUC when Yi is a Bernoulli presence/absence, and their time-dependent versions when Yi is a Poisson count (i.e., time-to-event late effects outcomes). In addition, we will consider “calibration” and “accuracy” aspects, as they are relevant in our prediction context.

#### Feature/pattern engineering

For the GLM with Elastic Net regularization, as is the case with any statistical modeling, we must prepare the set of candidate predictors before the modeling activity. This is particularly important for symptoms and other PGHD data collected longitudinally over 4 months at 28 time points (4 months x 7 consecutive days/month). Due to the complexity of the longitudinal data, we anticipate that this pre-modeling “feature engineering” will require a substantial amount of time and analytic efforts, but will offer less possibility of unexpected discovery. Initially, we will analyze each survivor’s 28 time points data on 20 symptoms, assessing consistency/variation of symptom existence and severity within each week and across 4 months, by sex, paying attention to the expected correlation across the 20 symptoms, particularly symptoms of the same domain. By comparing these patterns across 500 survivors in the training dataset, we will derive symptom patterns that characterize and differentiate subgroups of survivors. Similarly, we will analyze each type of other PGHD per survivor first, followed by the across-survivor comparisons, to derive patterns that characterize and differentiate survivor subgroups. We will use a combination of unsupervised learning methods (e.g., cluster analysis, projection pursuit), expert knowledge (e.g., known aspects/patterns of physical activities that affect QOL), and consideration of subgroup sizes.

#### Sample size consideration

For training purposes, we assessed power of identifying associated factors in predicting an outcome in the training dataset. For testing, we assessed the precision of sensitivity and specificity with the testing dataset. We focused on the time-to-event onset of/worsening chronic health conditions (Aim 2); the continuous outcomes (QOL scores & changes in Aim 1, and physical performance deficits in Aim 2) and the binary outcomes (unplanned healthcare utilization in Aim 2) would have higher power due to the continuous nature and higher expected prevalence. We calculated the minimum detectable hazard ratios with N=500 for outcomes with 10, 20, and 30% cumulative incidence in the training set, with 80% power and a Type I error probability of 0.05, using the power calculation approach for the proposed time-to-event analysis. The minimum detectable hazard ratio is 2.55 or smaller unless the outcome’s cumulative incidence is ≤10% and the predictor’s prevalence is ≤25%, indicating good power for a wide range of moderately predictive predictors. The testing data will include 100 subjects. Based on the precision of AUC from the equivalence to Wilcoxon statistic, 89 the margin of error of AUC estimation for underlying AUC of 0.90 is 0.12, 0.09, and 0.07 for 10%, 20%, and 30% cumulative incidence of chronic health conditions. For AUC of 0.80, it is 0.15, 0.11, and 0.09, providing sufficient precision in testing data.

## Discussion

The main objective of this study is to enable regular monitoring of PGHD to develop survivor-specific risk prediction models for adverse outcomes of childhood cancer and integrate this information into the existing EHR to improve survivorship care and outcomes. This novel approach leverages mHealth technology to assess daily symptoms, in combination with wearable devices/sensors, to assess momentary PGHD from typical daily activities, providing an opportunity to develop individualized risk prediction scores of impaired health status. The utilization of personalized risk prediction scores, coupled with enhanced communication between healthcare providers and survivors, holds the potential to enhance the standard of survivorship care and outcomes by early detection and timely intervention.

The use of digital health technologies for PGHD platform development and integration into an EHR could face some challenges. This study relies on participants’ access to mobile smartphones and access to a reliable data plan or stable internet connection. However, approximately 22% of Americans living in rural areas lack access to broadband internet compared to 1.5% of Americans living in urban areas (61, 62). Although innovation of mHealth technology is fast-paced, regulatory guidelines are still limited (63). Digital health technology guidelines have been drafted (but not finalized) by the U.S. FDA, and these guidelines describe clinical risks and privacy-related risks (64). Therefore, potential risks have been thoroughly described in the informed consent for the Health Share Study. Additionally, it is important to consider the population’s digital literacy. During the educational visit of the Health Share Study, a team member will thoroughly discuss the technology and activities that are required to improve the participants’ digital literacy as needed. The study team is also available for technical support throughout the study period. Another potential challenge is interoperability. The Health Share Study uses different systems (patient interface system, WHOOP, iHealth, EHR, etc.) that can exchange information in a secure format and merge cohesively for data integration and analysis. In addition to interoperability, data security is essential. The patient interface system is housed on a secure server with minimal risk of data loss. When working with outside companies, such as WHOOP and iHealth, the study team has created anonymous accounts for the users so that their information is protected. Finally, data acquisition for daily activities is vital to the analyses. We have set up a comprehensive reminder system to reduce missingness and increase compliance with the daily study activities.

Conventional approaches to predicting adverse events for survivors of childhood cancer rely heavily on treatment modality and regular follow-up in survivorship clinics; however, the Health Share Study promotes the novel approach of utilizing mHealth technology to collect daily biometric data and symptom reports from the home setting and before the clinic visits to improve health status monitoring and prediction. Rural populations are more likely to have additional comorbidities, poorer general health and an elevated risk of cancer-related mortality compared with non-rural populations (65, 66). Utilizing digital devices rather than relying solely on face-to-face medical assessment and diagnostic screening may increase access to care for rural communities to shorten the diagnosis interval. Additionally, through the third aim of this study, a dashboard will be created, as part of the patient portal, to display the predicted risk of adverse health outcomes for each participant. For future clinical applications, the dashboard will display levels of risk factors collected from the first four months that contribute significantly to future adverse health outcomes. This dashboard will also provide decision support for clinicians, allowing for timely bidirectional communication between survivors and clinicians.

### Future directions

Utilizing mHealth may decrease health disparities or improve equity for underserved populations/areas (67, 68). Determining how to best utilize mHealth technology and disseminate this technology into different areas is important to the future of cancer survivorship care. Following the Health Share Study, the investigators aspire to collaborate with medical centers to create and provide a package for other centers to adopt. This will require adapting the current procedures to implement the practices in different settings and evaluate the yield of this mHealth approach through a multicenter-based randomized controlled trial. Additionally, the investigators plan to develop a clinically actionable interventional strategy based on the findings to adapt modifiable behaviors to prevent adverse health outcomes for aging childhood cancer survivors. Finally, while this study focuses on the setting of hospitals/cancer centers that have EHRs to support cancer research and survivorship care, survivors may see primary care providers in community clinics rather than in cancer hospitals/centers for survivorship care. Community clinics, especially in rural or underserved areas, may not have the full functionality/capability of HERs, and the design of alternative methods is needed for implementing PGHD-based data collection and risk prediction.

## Data availability statement

All relevant data is contained within the article: The original contributions presented in the study are included in the article/supplementary material, further inquiries can be directed to the corresponding author/s.

## Ethics statement

The study involves human participants and was reviewed and approved by the Institutional Review Board at St. Jude Children’s Research Hospital. The participants provided their written informed consent to participate in this study.

## Author contributions

KH: Data curation, Methodology, Project administration, Writing – original draft, Writing – review & editing. MS: Data curation, Project administration, Writing – review & editing. AS: Writing – review & editing. KR: Data curation, Project administration, Writing – review & editing. IO: Data curation, Methodology, Project administration, Writing – review & editing. DT: Data curation, Writing – review & editing. SL: Data curation, Writing – review & editing. CW: Writing – review & editing. CC: Writing – review & editing. WM: Writing – review & editing. CV: Writing – review & editing. ME: Investigation, Writing – review & editing. SD: Investigation, Writing – review & editing. GA: Investigation, Resources, Writing – review & editing. KN: Data curation, Funding acquisition, Investigation, Resources, Writing – review & editing. MH: Data curation, Funding acquisition, Investigation, Resources, Writing – review & editing. YY: Conceptualization, Data curation, Funding acquisition, Investigation, Methodology, Resources, Supervision, Writing – original draft, Writing – review & editing. I-CH: Conceptualization, Data curation, Funding acquisition, Investigation, Methodology, Project administration, Resources, Supervision, Validation, Writing – original draft, Writing – review & editing.
